# Complications and Disasters After Minimally Invasive Tissue Augmentation with Different Types of Fillers: A Retrospective Analysis

**DOI:** 10.1007/s00266-021-02691-9

**Published:** 2021-12-22

**Authors:** Alperen S. Bingoel, Khaled Dastagir, Lavinia Neubert, Doha Obed, Thurid R. Hofmann, Nicco Krezdorn, Sören Könneker, Peter M. Vogt, Tobias R. Mett

**Affiliations:** 1 Department of Plastic, Aesthetic, Hand and Reconstructive Surgery, Burn Centre, Hannover Medical School, Carl-Neuberg-Str. 1, 30625 Hannover, Germany; 2 Institute of Pathology, Hannover Medical School, Carl-Neuberg-Str. 1, 30625 Hannover, Germany; 3 Department of Plastic, Aesthetic, and Reconstructive Surgery, Evangelical Hospital Göttingen-Weende, An der Lutter 24, 37075 Göttingen, Germany

**Keywords:** Aesthetic surgery, Injectable fillers, Filler complications, Permanent fillers, Self-injection, Silicone, Vaseline

## Abstract

**Background:**

The interest in youthful appearance and rejuvenating procedures is unbroken in our society. Besides surgical procedures, permanent fillers are utilized. The incorrect and unprofessional use of these substances, auto-injections in particular, have devastating results for patients and are challenging for the plastic surgeon. The aim of this retrospective study was to delineate the differences between permanent and non-permanent filler complications and appropriate treatment options.

**Methods:**

We conducted a retrospective study and researched the hospital information system in the time period from 2001 to 2020. Patients with unprofessional use of permanent fillers, auto-injections and injections of unformulated substances were determined. Age, gender, localization, complications, length of hospital stay, comorbidities, histopathological workups and surgical salvage procedures were noted. Descriptive statistics were calculated.

**Results:**

Seventeen patients were identified from 2001 till 2020. In four cases, auto-injections by the patients were the cause, whereas in the other patients the injections were performed by medical staff. Ages range from 18 to 57 years. Fourteen patients were female and three were male. The injected substances could be recognized as synthol, silicone, vaseline, fat tissue, hyaluronic acid as well as non-medical substances. Surgical procedures were necessary in eleven cases. One patient died because of the underlying diseases.

**Conclusion:**

Our results indicate different sequels of filler materials injected in an unprofessional way, possible complications, conservative and surgical techniques to resolve these rare complications. We suggest a staged therapy adjusted to the clinical symptoms. Milder symptoms can be handled conservatively, whereas severe infections, skin breakdowns or persistent granuloma are justifying indications for surgical treatment.

**Level of Evidence V:**

This journal requires that authors assign a level of evidence to each article. For a full description of these Evidence-Based Medicine ratings, please refer to the Table of Contents or the online Instructions to Authors www.springer.com/00266.

## Introduction

The demand for youthful appearance, a muscular body composition and appealing sexual characteristics is unbroken in modern society. Besides surgical procedures, different types of soft tissue fillers are favored by the patients and also the practitioners. They promise direct effects and fast recovery times. Fillers, whether permanent or not, have been on the rise in the last years according to the statistics of the International Society for Aesthetic Plastic Surgery (ISAPS) [1].

A common and frequently used substance is hyaluronic acid. Generally, it is proven by different research articles to be a safe and biocompatible filler material [2, 3]. Moreover, other materials are injected as well, e.g., paraffin, silicone, petroleum-jelly-based products, synthol and other fatty agents for skin care, which are freely available in retail. The incorrect and unprofessional use of resorbable and non-resorbable substances, particularly auto-injections by laypeople, have devastating results for patients e.g., tissue breakdown, necrosis and severe inflammation [4]. Auto-injections with exogenous non-medical substances increase these risks dramatically.

While swelling or bruising of injected regions with hyaluronic acid can be handled conservatively with cooling, steroids and antibiotics, wounds from permanent filler materials need more attention. These tissue reactions are maintained by an inflammatory process in response to non-authorized materials. Recurrences are often seen because of the remaining particles in the tissue. Common methods of choice in the acute phase are excision and debridement of the inflamed tissue and the substances. Control of the inflammatory process should be the main priority. Reconstruction is often planned as multiple-stage procedures when primary or secondary closure is not possible.

The aim of this retrospective study was to delineate the differences between permanent and non-permanent filler complications, treatment options as well as salvage strategies in severe cases of filler complications. A detailed analysis of histopathological features and the used materials was included.

## Patients and Methods

We conducted a retrospective study and researched the hospital information system (SAP, Walldorf, Germany) in the time period from 2001 to 2020 at the Department of Plastic, Aesthetic, Hand and Reconstructive Surgery. Inclusion criteria were the use of different types of filler materials as a cause for secondary intervention in our department.

The data of all patients that received professional and unprofessional use of filler materials, auto-injections and injections of unformulated, non-authorized substances regardless of the anatomical region were collected and included in this study.

The epidemiologic data comprised age as well as gender. Furthermore, the medical records of the patients were reviewed for affected anatomical localization, surgical procedures, complications, comorbidities, histopathological workups, type of injection, formula of the injected material, length of hospital stay and outcome.

Collection and processing of the data were carried out with Microsoft Excel (Microsoft Inc., Redmond, WA). Statistical analysis was performed using Prism 9.0 (GraphPad, La Jolla, CA). Descriptive statistics (mean and standard deviation) were used for the presentation of the provided data.

The present study was approved by the ethics committee of our institution with the approval number 8982_BO_K_2020 and was in line with the ethical standards of the Declaration of Helsinki and its later amendments and comparable ethical standards.

## Results

From 2001 to 2020, 17 patients were identified with the use of different types of filler materials. Table 1 presents the complete medical records of all patients included in this study. The mean age distribution was calculated with 40.9 ± 12.5 years (range 18–57).

**Table 1:** Medical records of patients with filler complications

Patient	Sex	Age	Diagnosis	Surgical Therapy	Complications	Comorbidities	Histopathology	Auto-injection	Formula	Length of stay	Outcome
1	w	41	Granuloma after lipofilling due to midface trauma right facial and periorbital region, radix of the nose	Excision of granuloma and primary closure	None	None	Granulating inflammatory reaction	No	Adipose tissue	2	Healed
2	w	37	Foreign body inclusions in the face after injection of foreign filler and lipofilling	Excision of foreign body granuloma left periorbital area	1x recurrent granuloma	None	Presumable hyaluronic acid	No	Unknown	1	Healed
3	m	51	Severe soft tissue defects in both thighs, right hip and groin due to spontaneous perforation of granulomas after injection of paraffin/silicone	Multiple excisions and debridements, lavage and tamponade with iodine gauze	Death	Terminal stage of AIDS Hepatitis B Tuberculosis	Amorphous foreign material with granulocytic inflammation	No	Paraffin/silicone	23	Death due to underlying disease
4	m	37	Intramuscular foreign body granuloma in biceps and triceps after synthol injections	Therapy rejected by patient	None	Nicotine abuse Opiate dependency	None	Yes	Synthol	0	Healed
5	w	57	Granuloma in both lips after silicone injection	Excision of granuloma in both lips	1x recurrent granuloma	None	Severe foreign body reaction with chronic fibrotic inflammation and hyperplasia of squamous epithelium of both lips	No	Unknown	3	Healed
6	m	40	Painful deformity in penis shaft after infection of unknown material	Excision of granuloma	None	None	Prominent foreign body reaction and chronic sclerosing inflammation	Yes	Unknown	10	Healed
7	w	20	Local swelling and bruising of both lips	None	None	Depressions Borderline personality disorder	None	Yes	BCN Adipo Forte	0	Healed
8	w	40	Painful lipogranuloma thorax, abdomen and both flanks after injection of paraffin for augmentation mammoplasty	Multiple excisions of lipogranuloma thorax, both flanks and breasts, abdomen	3x recurrent lipogranuloma	None	Sclerosing and granulomatous inflammation, numerous foreign body giant cells	No	Paraffin	7, 6*, 4*, 3*	Recurrent lipogranuloma
9	w	57	Painful granuloma in zygomatic arch and lower lip	Excision of foreign body granuloma zygomatic arch and lower lip	1x recurrent granuloma	Esophageal cancer	Foreign body granuloma	No	Dermalive	1	Healed
10	w	18	Necrotizing phlegmon of the upper lip	Incision and evacuation of foreign material, debridement, primary closure with drains	None	None	Necrosis of muscle and adipose tissue	Yes	Vitamin E oil	6	Healed
11	w	56	Local swelling and bruising of the right face and both lips	None	None	Glucogenosis	None	No	Juvederm	0	Healed
12	w	44	Infected granulomas in both lips	Incision and evacuation of infection, primary closure with drains	1x recurrent infected granuloma	None	None	No	Unknown	0	Healed
13	w	47	Local swelling and bruising of both nasolabial folds and glabella	None	None	None	None	No	Unknown	0	Healed
14	w	18	Spontaneous drainage of an upper lip abscess	None	None	Acne vulgaris	None	No	Stylage	0	Healed
15	w	44	Painful nodules periorbital region and cheeks	None	None	None	None	No	Juvederm	0	Healed
16	w	38	Necrosis of brow ridge	None	None	None	None	No	Unknown	0	Healed
17	w	50	Granuloma formations with tissue breakdown left lower leg	Multiple excisions and debridements, NPWT, split skin grafts	None	Pancreatic carcinoma	Granulating inflammatory reaction	No	Paraffin/silicone	34	Healed

In terms of gender, the distribution showed 82.35% (*n* = 14) female and 17.65% (*n* = 3) male patients. The anatomical localizations are shown in Table 2. The lips (*n* = 7) were the most common site for injection of filler materials followed by the periorbital region (*n* = 3).

**Table 2:** Overview of areas injected by fillers

Face	Periorbital region Temporal region Lips Nasolabial fold Glabella/brow ridge Cheeks	*n* = 3 *n* = 1 *n* = 7 *n* = 1 *n* = 2 *n* = 1
Trunk	Breasts	*n* = 1
Upper extremity	Upper arm	*n* = 1
Lower extremity	Thighs, hips, lumbar areas	*n* = 2
Sexual organs	Penis	*n* = 1

Surgical therapy was necessary in 11 of 17 (65.70%) patients. One of these patients rejected surgical therapy.

In six patients, the treatment was successful with conservative methods including orally administered antibiotics. These six patients had been injected with hyaluronic acid.

In the surgically treated ten cases, the regimen consisted of radical excision or incision, debridement and primary closure or secondary wound healing. In seven patients, one single surgical procedure was sufficient. One patient had continuous problems because of recurrent granulomas. In one patient, open wound therapy was necessary due to ongoing inflammation.

No recurrent complications were evident in ten patients after the initial treatment in our department. Six patients suffered from recurrent granulomas. In four cases, a second radical excision with primary closure was successful. One patient is still in therapy with surgical procedures performed once a year, because of spread siliconoma. One patient died because of a depleted immune system without any sign of wound healing and malnourished status suffering from acquired immune deficiency syndrome (AIDS) in the final stage.

In four cases, the injections were performed by the patients themselves. In the other 11 patients, the injections were performed by medical professionals, and in one patient by an alternative medicine practitioner.

**Fig. 1:**
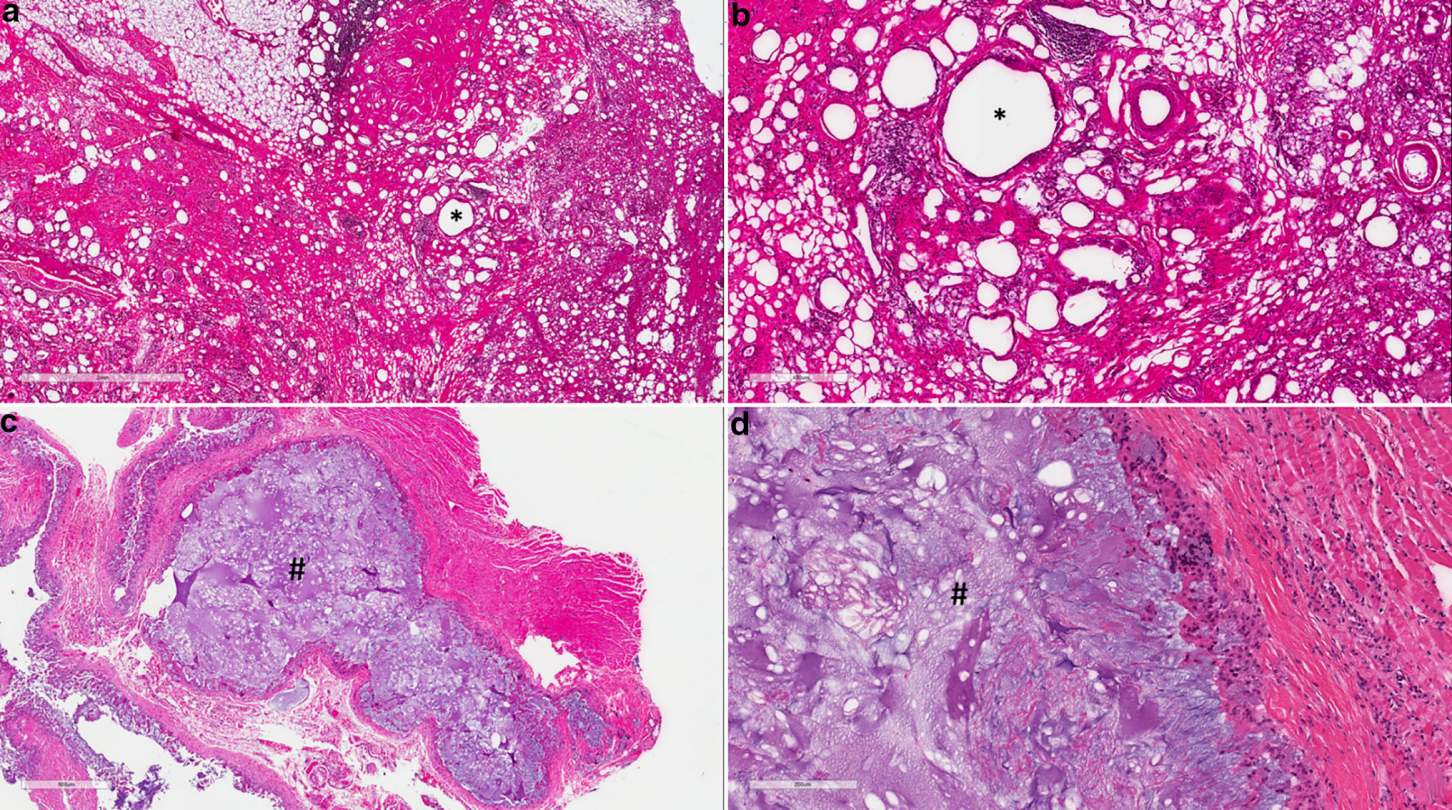
Histopathology of paraffin injection (**a** and **b**) and hyaluronic acid injection (**c** and **d**) Adipose tissue, interspersed with alternating cellular fibrosis areas with small fibrotically demarcated oil cysts (*) with accompanying calcifications, lymphohistiocytic infiltrates and sometimes grouped multinucleated giant cells of foreign body type (**a** and **b**). Soft tissue with abundant, non-anisotropy foreign material (#) and numerous surrounding multinucleated giant cells and histiocytes, partially encapsulated (**c** and **d**). Scale bars **a** 2mm, **b** 400µm, **c** 900µm and **d** 200µm

Histopathological workups were performed in nine cases with various findings that showed inflammation, foreign body inclusions and granulomas (Fig. 1). The histopathologic workup in two patients pointed out the injection of hyaluronic acid as well.

The injected substances could be identified subsequently as hyaluronic acid from different trademarks, synthol, paraffin, silicone, petroleum jelly, adipose tissue and vitamin E oil. Three cases are presented in depth below.

Nine patients were treated in-house; the other eight patients received ambulatory treatment. The mean length of the nine inpatients accounted for 10 ± 11.4 days (range 1–34) without readmissions.

Table 2 shows the different sites of injection.

### Case report 1

**Fig. 2:**
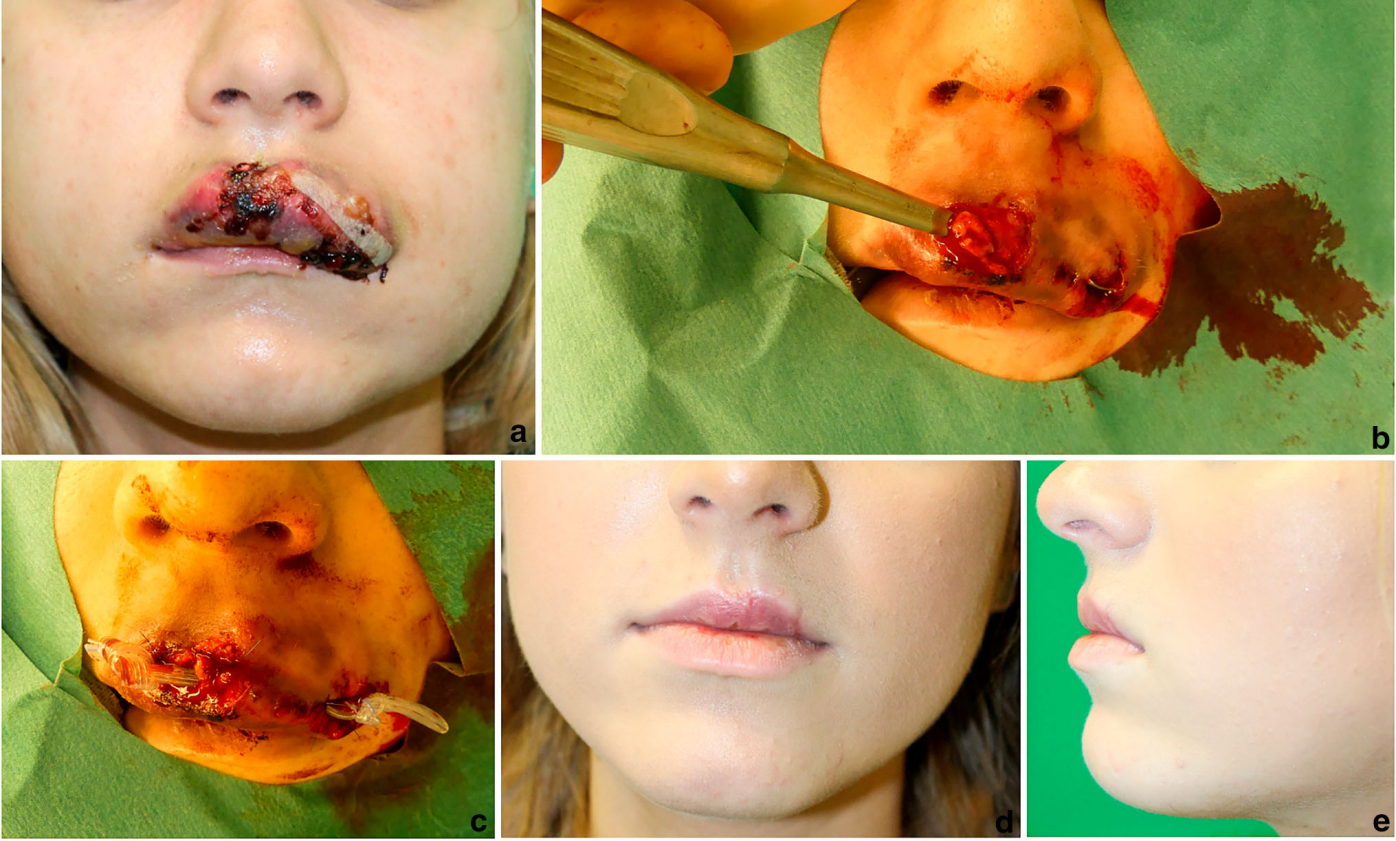
Clinical course from the initial presentation till full recovery: **a** shows the initial presentation of the patient with beginning inflammation in the upper lip region after injection. **b** Presents the intraoperative finding. A sharp curette can be inserted almost fully into the upper lip. **c** Displays the direct postoperative result with a partial closure and small plastic tubes. **d** and **e** Show the postoperative results after full restitutio ad integrum with an aesthetically satisfactory result

The clinical course of an 18-year-old woman who presented in our emergency room with a necrotizing phlegmon of the upper lip was noteworthy. She auto-injected a non-medical formula of vitamin E oil into her upper lip for augmentation purposes. The upper lip was severely inflamed with tissue breakdown and purulent exudate (Fig. 2a). We decided to perform multiple incisions with debridement of inflamed tissue and the foreign material. The wound was partially closed with small tubes (Fig. 2b, c). Additionally, intravenous antibiotics (amoxicillin and clavulanic acid) were administered for 5 days, the tube was removed on the 2nd postoperative day, and secondary healing was intended. Figure 2d, e outlines the pleasant outcome after almost 6 weeks postoperatively.

### Case report 2

**Fig. 3:**
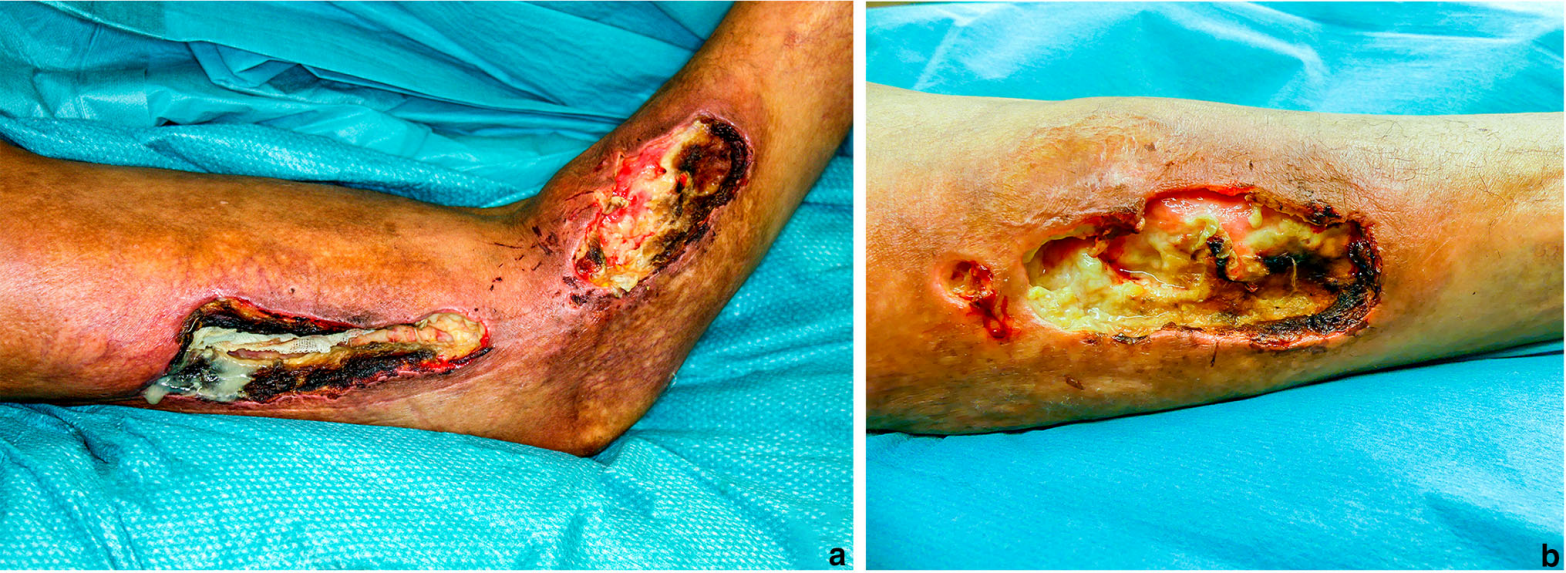
**a** and **b** Present the severe tissue breakdown with necrosis and inflammation of the thigh and lower leg area of the second case before debridement due to paraffin injections in the gluteal area in the second case report

The second patient was a 51-year-old transsexual woman with paraffin injections almost 20 years ago in the gluteal area. This led to recurrent granuloma formations with spontaneous perforation in both legs, hips and lumbar area with massive tissue breakdown and wounds (Fig. 3a, b). We performed multiple debridements and favored open wound therapy. Because of a compromised immune system based on end-stage AIDS, the patient died due to pulmonary infection.

### Case report 3

**Fig. 4:**
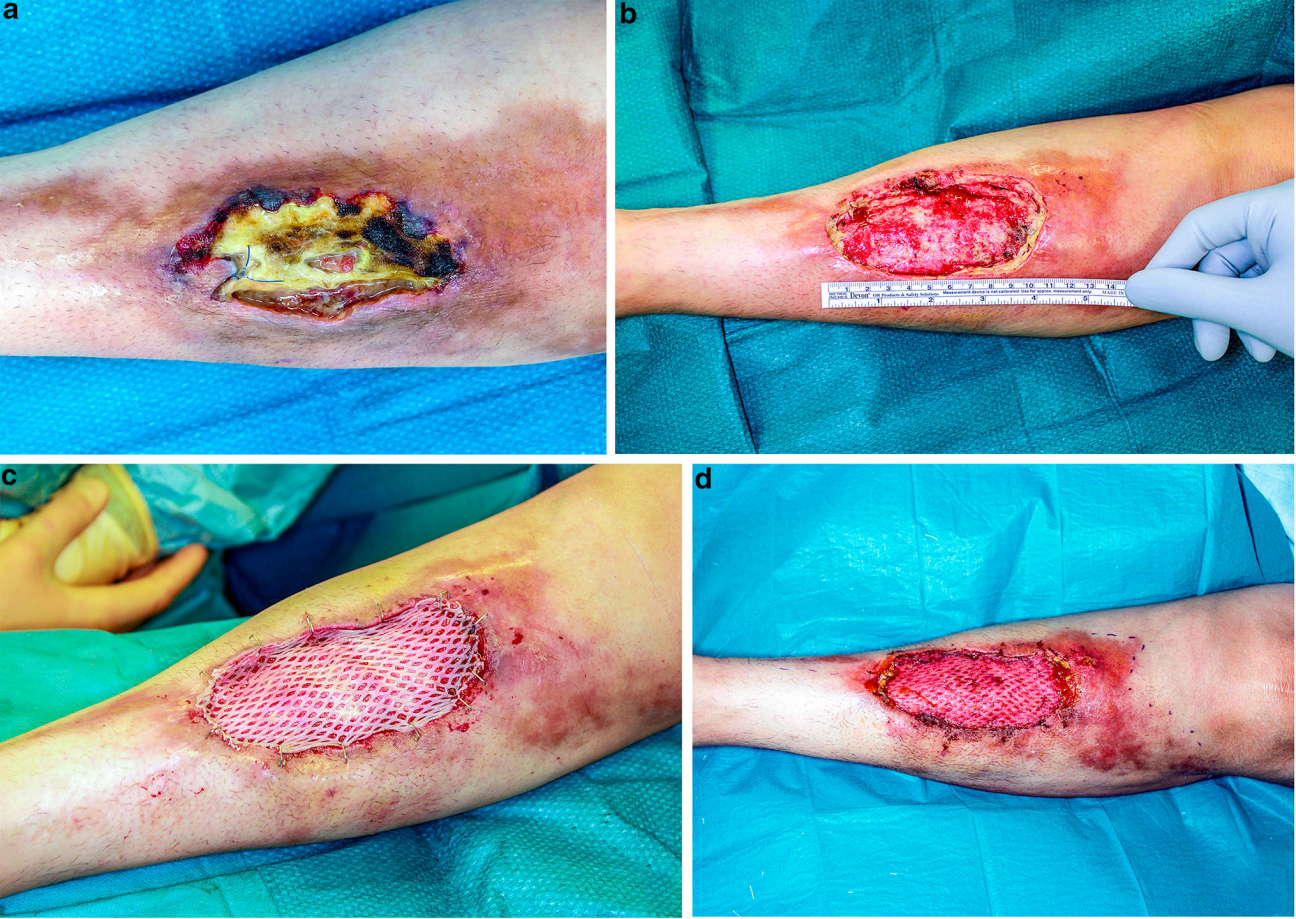
**a** Displays the preoperative wound situation of the third case with necrosis and silicone running out of the wound. The injections were performed 20 years ago. After several debridements and negative wound pressure therapy (**b**), split skin grafts were used to cover the wound (**c**). **d** Shows the postoperative result

The third patient was a 50-year-old woman with a chronic wound on the left lower leg. This occurred after silicone injections in the gluteal area about 20 years earlier. There was no history of treatment for the wound. We performed several debridements and negative wound pressure therapy to condition the wound (Fig. 4a, b). Histopathological workups showed granuloma formations based on silicone. The wound was successfully covered with split skin grafts, and the patient could be discharged (Fig. 4c, d).

## Discussion

In this study, we describe a heterogeneous cohort of patients that had received injections of different types of filler materials for aesthetic purposes. The patients were treated for painful and inflamed granuloma formations surgically in 10 of 17 cases. Six patients were treated conservatively in a successful manner, and one died because of the preexisting condition of the final stage of AIDS.

The face was the most common region that was subject to injections with two having been auto-injected. In total, we observed four cases with auto-injections.

The treatment of the face with filler materials like hyaluronic acid requires meticulous planning, proper technique and understanding of the anatomical structures. The literature provides plenty of studies in order to reduce the risks and improve safety, particularly using filler materials in facial danger zones [5–7], even though case reports with severe complications were described [8].

In the hands of professionally trained medical staff, hyaluronic acid is a safe and biocompatible filler material [3]. The severity of the complications that were observed in this cohort study like local bruising and swelling is, generally speaking, in line with the data in the literature and can be handled conservatively [9]. Nevertheless, the vascular anatomy of the face is complex and rich of variations. This fact also applies to the anatomy of the lips [10]. Even a small amount of filler material can have a big impact on the aesthetic result [11]. Auto-injections of non-medical formula in the face by laypeople without any knowledge can have fatal consequences as we demonstrated in one of our cases. Wolfram et al. reported good functional and aesthetic results of surgical intervention in these cases, if conservative treatments failed [12].

Paraffinomas of the breast are a well-known complication after augmentation mammoplasty with paraffin [13]. This filler material was used widely by unqualified people in the 1950/1960s in China and Europe as well. The injections resulted in grotesque appearances, severe soft tissue damage and sometimes even culminated in mastectomy [14]. Ho et al. described in their study a destruction of the anterior chest wall with ongoing ulcerations and residual multiple paraffinomas in one patient. This description is consistent with the observation in two of our cases. Paraffin provokes tissue inflammation, and despite radical debridement, it seems to remain in the tissue with migration into the adjacent parts of the body like the abdominal wall and even down to the lower extremities. This causes hard nodules, abscesses, fistulas and skin destruction [15]. Di Benedetto et al. delineated a cohort study of 26 patients with paraffinomas and suggested that the wide surgical excision should be the key procedure in the therapy of paraffinomas in the breast and trunk region. In this context, histopathological workups and imaging techniques like MRI or mammography have evidence in cases with ambiguous clinical findings in order to rule out other diseases [16, 17].

The largest case study with a total of 173 patients was published by Park et al. [18]. In total, 121 patients received perilesional surgical excision of the foreign body granulomas with a higher satisfaction rate than the patients who received therapy with steroids or hyaluronidase. Park et al. emphasize the importance of proper patient selection for a successful outcome when favoring a perilesional surgical approach [19].

Auto-injections of foreign materials like paraffin and mineral oils into the penis were described to increase girth of the penis by laypeople in various case reports [20–23]. This can even result in a Fournier’s gangrene with catastrophic results [24].

Rosellen et al. recently published treatment strategies for paraffinomas of the penis [25]. For smaller lesions of the penis shaft, they recommend local excision with primary closure which is in line with our treatment strategy in this study. In case of larger lesions with severe inflammation, excision and skin transplantations up to local flaps are feasible options.

Synthol, which we found in one of our patients, is commonly used in the bodybuilding scene to enhance muscular appearance [26–30]. The formula comprises 85% oil (medium-chain triglycerides), 7.5% lidocaine and 7.5% alcohol [27]. The injection is followed by immediate increase in muscle appearance. Complications are lipogranuloma due to the high percentage of oil and progressive tissue destruction [26]. In these cases, radical surgical excision seems to be the only valuable therapy.

Another filler substance that may be found in patients is polymethylmethacrylate (PMMA). PMMA can also lead to granuloma formation. Park et al. demonstrated in 13 patients that both, surgical excision and steroid injections, can be a valuable therapeutic option [31].

Auto-injections of filler materials seem to be an “underreported phenomenon” [4], since there are just some case reports available [32–34]. Even though the unrecorded cases of auto-injections might be much higher, another potential reason might be the costs for secondary treatment which are not covered by any health insurance. Since auto-injections and laymen treatments might be a price-driven decision, the following professional treatment has an economic burden on the patients.

Furthermore, hyaluronic acid as a filler material can be purchased in many countries without prescription. This opens the door for auto-injections or injections by laypeople and underlines the necessity for legal control of these substances.

Fat grafting and transfer into scar regions is a reconstructive strategy that gained attention in the last years due to supporting evidence [35–39]. However, late inflammatory changes of adipose tissue after lipofilling into scars—like in one of our cases—were rarely reported [40, 41]. Sa et al. reported nine patients with lipogranuloma and inflammatory reactions after autologous fat injections (42). Six patients required surgical excisions. Although lipofilling for reconstructive purposes seems to be a safe procedure, surgeons should be aware of complications like inflammatory lipogranuloma.

In summary, a staged therapy with watchful waiting and topical cooling, steroids and antibiotics are applicable in milder symptoms. Severe infections, skin breakdowns or persistent granuloma are justifying indications for surgical treatment. In dependence of the soft tissue quality and wound situation, a partial closure or open wound therapy might be the best choice. Even in secondary healing cases, an aesthetically reasonable result might be achieved or reconstructed by secondary corrections.

Limitations of this study are mainly the retrospective nature of the study design and the small number of cases. This caused a lack of traceability in some of our cases about the used materials and techniques. Nevertheless, this study can help clinicians to overview different types of filler materials, possible complications and conservative and particularly surgical techniques to resolve these rare complications.

## Conclusion

Aesthetic treatments with permanent and non-permanent filler materials have been on the rise lately. Patients and clinicians should be aware of possible complications like foreign body granuloma beside milder symptoms like bruising and swelling. There seems to be supporting evidence that surgical excision is a safe and effective therapy, especially when non-medical fillers were used with severe complications. Furthermore, we deem it justified to require prescription for filler materials that are currently freely available.
